# Integrated machine learning–based RNA sequencing and single-cell analysis reveal RNA methylation regulation patterns in the immune microenvironment of Alzheimer’s disease

**DOI:** 10.4103/NRR.NRR-D-24-01650

**Published:** 2025-09-03

**Authors:** Shuguang Wu, Ting Guo, Xingyongpei Zheng, Caihong Gu, Yujie Hu, Xinru Gu, Xinyu Zhou

**Affiliations:** 1 Department of Anesthesiology, The Second Affiliated Hospital, Zhejiang University School of Medicine, Hangzhou, Zhejiang Province, China; 2 Department of Geriatric Medicine, Shanghai Sixth People’s Hospital Affiliated to Shanghai Jiao Tong University School of Medicine, Shanghai, China; 3 Department of Neurology, The Affiliated Lianyungang Hospital of Xuzhou Medical University, Lianyungang, Jiangsu Province, China; 4 Department of Neurology, The First Affiliated Hospital of Kangda College of Nanjing Medical University, Lianyungang, Jiangsu Province, China; 5 Department of Neurology, Nanjing Drum Tower Hospital, Affiliated Hospital of Medical School, Nanjing University, Nanjing, Jiangsu Province, China; 6 Department of Neurology, Lianyungang Clinical College of Nanjing Medical University, Lianyungang, Jiangsu Province, China

**Keywords:** Alzheimer’s disease, immunity, long non-coding RNAs, machine learning, nerve regeneration, risk model, RNA methylation

## Abstract

Alterations in RNA methylation may affect the initiation and development of Alzheimer’s disease. However, the exact nature of the relationship between RNA methylation and Alzheimer’s disease remains unclear. In this study, RNA methylation levels were analyzed by bulk transcriptomic and single-cell RNA sequencing. The expression levels of RNA methylation regulators were confirmed using molecular biology techniques. Co-expression network analysis was used to identify relevant long non-coding RNAs. Molecular subtypes related to RNA methylation were classified, and variations in clinical characteristics, biological behavior, and immune signatures between subtypes were assessed. Machine learning approaches were applied to identify methylation-associated long non-coding RNAs, which were used to construct a risk model and nomogram for Alzheimer’s disease. Potential therapeutic agents for different risk groups were predicted, and *in vitro* experiments were conducted to identify key RNA methylation events. Single-cell analysis demonstrated enhanced RNA methylation in patients with Alzheimer’s disease, particularly within T cells, B cells, and NK cells. Quantitative reverse transcription-polymerase chain reaction and western blot confirmed alterations in RNA methylation regulators in neurons treated with amyloid-β oligomers *in vitro*. This evidence supported the classification of patients with Alzheimer’s disease into heterogeneous subtypes. Specifically, subtype 1 was identified as the immune-active subtype, while subtype 2 was characterized by a metabolic phenotype. Machine learning algorithms identified five significant methylation-associated long non-coding RNAs —LINC01007, MAP4K3-DT, MIR302CHG, VAC14-AS1, and TGFB2-OT1—that accurately predict clinical outcomes for patients with Alzheimer’s disease. These patients were classified into low- and high-risk categories; the latter group displayed higher immune infiltration, upregulated immune regulatory gene expression, and elevated immune scores and responded better to treatment with arachidonic-trifluoroethane. These findings suggest that dysregulated RNA methylation alters the immune microenvironment in Alzheimer’s disease and is closely associated with its progression. This phenomenon provides novel insights into potential therapeutic strategies for Alzheimer’s disease that target RNA methylation.

## Graphical abstract

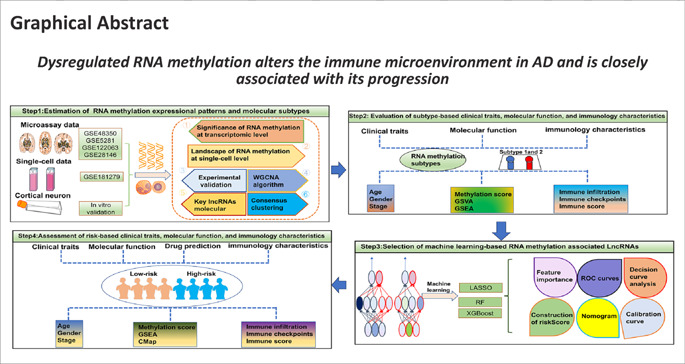

## Introduction

Alzheimer’s disease (AD), which is the primary cause of dementia, is rapidly becoming one of the most costly and fatal neurological conditions (Scheltens et al., 2021). In the past few decades, there have been noteworthy developments in pharmacotherapy for AD, including the development of cholinesterase inhibitors, N-methyl-D-aspartate (NMDA) receptor blockers, antidepressant drugs, and antipsychotic treatments (Briggs et al., 2016; Passeri et al., 2022). However, in practice, pharmacological treatments are not universally efficacious, which is mainly attributed to the heterogeneity of AD. Studies have shown that the heterogeneity of AD is usually manifested by distinct molecular characteristics (Kaszniak, 1988; Lambon Ralph et al., 2003; Canevelli et al., 2013). Research has established a significant connection between AD variability and the success rate of clinical treatments. Nevertheless, the molecular attributes contributing to this diversity in AD remain poorly defined.

RNA methylation is pivotal in modulating several cellular processes, including RNA export from the nucleus, translation, transcript splicing, and processing of non-coding RNA sequences (Han et al., 2023; Wang et al., 2023). The most widely studied methylation modification patterns are N6-methyladenosine, 5-methylcytosine, and N1-methyladenosine (Shi et al., 2020). One study reported that N6-methyladenosine- and N1-methyladenosine-mediated gene expression levels are closely linked to AD progression (Han et al., 2020). Additionally, a recent study has revealed differences in the expression levels of 5-methylcytosine RNA methylation modifiers, specifically nucleolar protein 2 (NOP2)/Sun RNA methyltransferase 6 (NSUN6) and NOP2/Sun RNA methyltransferase 7 (NSUN7), between individuals with AD and healthy controls (PerezGrovas-Saltijeral et al., 2023). These discoveries suggest that RNA methylation is a possible target for AD treatment. Nevertheless, the role and detailed mechanisms of RNA in AD pathology are largely undefined, and thus require in-depth investigation.

Long non-coding RNAs (lncRNAs) are transcripts that are over 200 nucleotides long and do not encode proteins. Acknowledged for their crucial involvement in biological processes such as cell division, differentiation, and energy metabolism, these RNA molecules are additionally linked to the onset of various diseases (Li et al., 2021; Nemeth et al., 2024). lncRNAs are associated with AD and are instrumental in modulating the expression of genes associated with the condition (Idda et al., 2018; Wu and Kuo, 2020; Chen et al., 2021; Lauretti et al., 2021). Consequently, lncRNAs may function as indicators for AD progression and are potentially feasible targets for therapeutic intervention. Currently, no evidence illustrates a connection between RNA methylation–associated lncRNAs and AD.

Single-cell RNA sequencing (scRNA-seq) is a technique used to study gene expression within individual cells. It has uncovered genes and pathways related to cognitive function, dementia, and adaptive responses to AD pathology, providing crucial insights into understanding the molecular mechanisms of AD and discovering potential new treatments (Wang et al., 2022; Jin et al., 2024). Machine learning is crucial in disease diagnosis, prognosis, and treatment within the medical field. Analyzing extensive medical data, including imaging scans, genetic details, and patient health records, significantly improves the accuracy and efficiency of diagnostic processes. Machine learning algorithms can detect patterns and biomarkers that might indicate the presence of diseases, including AD, often more reliably and at an earlier stage than might traditional techniques (Cai et al., 2023; Tao et al., 2024). Recent studies have shown that machine learning can be used to predict and diagnose AD (Lai et al., 2022; Yue et al., 2023). In this study, we used bulk and scRNA-seq, along with molecular biology techniques, to thoroughly explore the function and underlying mechanisms of RNA methylation in AD. This research will offer new perspectives for developing AD treatment strategies that target RNA methylation.

## Methods

### Data collection and preprocessing

Transcriptomic data from patients with AD were retrieved from the Gene Expression Omnibus (GEO) repository (https://www.ncbi.nlm.nih.gov/geo/). Three datasets (GSE48350, GSE5281, and GSE28146) that included both lncRNAs and mRNAs were identified (Liang et al., 2007; Berchtold et al., 2008; Blalock et al., 2011). Additionally, the GSE122063 dataset, primarily consisting of mRNAs, was selected (McKay et al., 2019). Detailed information about these datasets is presented in **Additional Table 1**. GSE48350 was designated as the training set, while GSE122063, GSE5281, and GSE28146 were chosen as the validation sets. The raw data were subjected to log2 transformation and subsequently normalized using the Robust Multi-Array Average (RMA) function of the affy R package (https://bioconductor.org/), followed by batch correction using limma’s normalize BetweenArrays function (https://bioconductor.org/). The limma package (https://bioconductor.org/) was used to identify differentially expressed lncRNAs using the false discovery rate method. Genes that were differentially expressed among subtypes or risk groups were defined by the following criteria: an absolute log2 fold change (|log2FC|) exceeding 0.5 and a false discovery rate threshold below 0.05.

**Additional Table 1:** The detailed information for gene datasets

GEO accession	Data type: data type	Number of AD	Number of Control
GSE181279	GPL24676:scRNA-seq	3	2
GSE167490	GPL24676:scRNA-seq	10	6
GSE48350	GPL570:bulk RNA-seq	80	173
GSE5281	GPL570:bulk RNA-seq	87	74
GSE122063	GPL16699:bulk RNA-seq	56	44
GSE28146	GPL570:bulk RNA-seq	22	8

AD: Alzheimer’s disease; GPL16699: Agilent-039494 SurePrint G3 Human GE v2 8x60K Microarray 039381 (Feature Number version); GPL24676: Illumina NovaSeq 6000 (Homo sapiens); GPL570: Affymetrix Human Genome U133 Plus 2.0 Array; scRNA: single-cell RNA.

Single-cell transcriptome data from GSE181279 and GSE167490 were obtained from the GEO database and adjusted using the NormalizeData function within the Seurat package (https://satijalab.org/seurat/) in R. Dataset integration and batch effect mitigation were accomplished using Seurat’s IntegrateData function. After integration, principal component analysis and t-distributed stochastic neighbor embedding (t-SNE) were conducted on the combined dataset. Cells expressing fewer than 200 or more than 5000 genes and those with mitochondrial gene expression exceeding 15% (GSE181279) or 10% (GSE167490) of total gene expression were excluded. Following this filtering step, 36,717 immune cells and 103,988 brain-specific cells were retained for further analysis. Variable features across the cells were identified using Seurat’s FindVariableFeatures, selecting the top 2000 genes with the most variable expression. Cell clustering was performed with the FindClusters function, revealing distinct cellular subpopulations. Immune-related cell types were annotated utilizing the Celltypist Python tool (https://www.celltypist.org/), following previously described methodologies (Domínguez Conde et al., 2022), and brain-specific cell types were annotated using known marker genes. Furthermore, we determined differentially expressed genes (DEGs) within individual cell clusters using the “FindAllMarkers” function, setting the log fold change threshold to 0.5 and the adjusted P-value to less than 0.05.

### Calculation of the RNA methylation score

A total of 47 RNA methylation regulators reported in previous studies (Xu et al., 2022; Zhao et al., 2022; Zou et al., 2022) were included in our study. The expression landscapes of these RNA methylation regulators were visualized by heatmap and a violin plot, and a gene relationship network diagram was constructed using graphing software to detect correlations. The relationships among RNA methylation regulators and 28 types of infiltrating immune cells were assessed by Spearman correlation analysis. RNA methylation scores based on the bulk transcriptome data were estimated using the single sample gene set enrichment analysis (ssGSEA) algorithm (https://www.genepattern.org/) (Charoentong et al., 2017). Single-cell transcriptome data-based RNA methylation scores were calculated using the “AUCell”, “UCell”, “singscore”, and “ssgsea” algorithms in the irGSEA package (https://github.com/chuiqin/irGSEA/).

### Primary culture of cortical neurons and construction of the Alzheimer’s disease model

Primary culture of cortical neurons was carried out in accordance with earlier research (Lai et al., 2020), with a few minor alterations. Briefly, pregnant Sprague–Dawley rats (gestational age 16–18 days; Jiangsu Huachuang Sino Company (Taizhou, Jiangsu, China)) were anesthetized by intraperitoneal injection with 4% sodium pentobarbital 40 mg/kg (Hmitech, Beijing, China), and the embryonic rats were removed. The cortical tissues of the embryonic rats were finely chopped and subjected to enzymatic dissociation with 0.25% trypsin at 37°C for 20 minutes. After trypsinization, the tissues were further chopped into smaller pieces. The trypsinized and minced tissue was then incubated at 37°C in neurobasal medium (Gibco, Grand Island, NY, USA). After an initial medium change 8 hours following seeding, the medium was replaced with fresh medium every 2–3 days. The cultures were maintained at 37°C in an environment with 5% CO_2_ for 7–9 days.

Oligomeric amyloid-β (Aβ)1–42 was prepared according to a previously published method (Dahlgren et al., 2002), with minimal modifications. One milligram of Aβ_1–42_ was dissolved in pre-chilled hexafluoroisopropanol (Sigma, St. Louis, MO, USA) to a final concentration of 1 mM Aβ_1–42_ and incubated at room temperature (25°C) for 30–60 minutes. Next, the solution was incubated on ice for 5–10 minutes. The supernatant containing the Aβ_1–42_-HFP peptide was removed and stored at –20°C. Finally, the supernatant was centrifuged (14,000 × *g* for 10 minutes at 4°C). to remove precipitates, and the purified supernatant containing the Aβ_1–42_ peptide was collected for subsequent analyses. To create an *in vitro* AD model, primary cortical neurons (day *in vitro* 7–8) were exposed to 20 μM Aβ_1–42_ oligomers at 37°C for 12 hours. Then, the culture medium was replaced with standard neurobasal medium. All procedures involving animals were approved by the Experimental Animal Center of Jiangsu Kangyuan Pharmaceutical Co., Ltd. (approval No. IACUC 2024073020) on July 30, 2024 and conducted in accordance with the National Institutes of Health Guide for the Care and Use of Laboratory Animals (8^th^ ed., National Research Council, 2011).

### Quantitative reverse transcription-polymerase chain reaction

Total RNA was extracted from primary cortical neurons using TRIzol reagent (Thermo Fisher Scientific, Waltham, MA, USA) according to the manufacturer’s instructions. RNA quantity, purity, and integrity were assessed by spectrophotometry and agarose gel electrophoresis. The RNA was then reverse-transcribed into cDNA utilizing a RevertAid First Strand cDNA Synthesis Kit (Thermo Fisher Scientific), oligo(dT) primers, and reverse transcriptase. The cDNA was then analyzed by quantitative reverse transcription-polymerase chain reaction (qRT-PCR) using an ABI 7500 Real-Time PCR system (Applied Biosystems, Foster City, CA, USA) and an qRT-PCR SYBR Green Kit (YEASEN, Shanghai, China). The reaction mixture was prepared by adding the forward and reverse primers specific to the genes of interest–YTH domain-containing protein 1(YTHDC1), YTH domain family 2 (YTHDF2), NSUN6, DNA-methyltransferase-3B (DNMT3B), and Fat mass and obesity-associated (FTO)–to the SYBR Green Master Mix, along with the cDNA template. The PCR primer sequences are listed in **Additional Table 2**. YTHDC1, YTHDF2, NSUN6, DNMT3B, and FTO mRNA expression levels relative to β-actin mRNA were calculated using the cycle threshold (ΔCt) method.

**Additional Table 2:** Quantitative reverse transcription-polymerase chain reaction primers targeting the related genes

Gene	Primer sequence (5’-3’)
YTHDC1	Forward: CATCCCTACTACCAGCACCAT Reverse: TTCTCCGACCACTGACAACG
YTHDF2	Forward: CTACAAGCACACCACTTCCAT Reverse: TTAGGATAAGCAGACGCAACC
NSUN6	Forward: ACCACAGATCGGAGGAGAAG Reverse: TCCAAGAGAGTTCGTGTCCAT
DNMT3B FTO	Forward: CCGATTCCTGGCATGTAACC Reverse: TGCTGTCCTACTGAACTCCAA Forward: CTGTGT TTTGGCTGGCTCAC Reverse: TCGCCATTGTCCGAGTCATT
β-Actin	Forward: TGAACCCTAAGGCCAACCGT Reverse: GTACGACCAGAGGCATACAGG

DNMT3B: DNA-methyltransferase-3B; FTO: Fat mass and obesity-associated; NSUN6: Nop2/Sun RNA methyltransferase; YTHDC1: YTH domain-containing protein 1; YTHDF2: YTH domain family 2.

The PCR cycling parameters included an initial denaturation stage at 95°C, followed by 40 cycles of a 15-second denaturation step at 95°C and 15-second annealing and extension step at 60°C. Fluorescence data were gathered after each extension step, and threshold cycle (Ct) values were ascertained using ABI 7500 software (Applied Biosystems). The software automatically determines the point where the fluorescence signal surpasses the background level. To achieve precise quantification, a standard curve was created by serial diluting a reference cDNA sample. The amplification efficiency of each primer pair fell within the 90%–110% range. The specificity of the PCR assays was confirmed by conducting a melting curve analysis conducted after the PCR protocol was complete. During this analysis, the temperature was gradually increased from 60°C to 95°C, with continuous fluorescence data collection. This step confirmed the presence of a single PCR product without primer-dimers or non-specific amplification. The Ct values of the target genes were normalized to the endogenous control gene β-actin using the ΔCt method to mitigate variations in cDNA quality and quantity. Relative gene expression levels were calculated by contrasting the ΔCt values of samples with those of a control or calibrator group, thereby determining the fold change in gene expression. All experiments were repeated three times.

### Western blot analysis

Total protein was extracted from primary cortical neurons using radio immunoprecipitation assay lysis buffer (Thermo Fisher Scientific), which effectively solubilizes cellular proteins while maintaining their integrity. The lysate was centrifuged to eliminate any insoluble fragments, and the supernatant containing the target proteins was collected. The protein concentration was determined using a bicinchoninic acid protein assay kit (Thermo Fisher Scientific) to ensure uniform protein loading. In total, 25 µg of protein from each sample was subjected to standard SDS-polyacrylamide gel electrophoresis (Bio-Rad, München, Germany). Following electrophoresis, the proteins were transferred to polyvinylidene fluoride membranes (Millipore, Bedford, MA, USA). The membranes were then incubated with the primary antibodies rabbit anti-FTO (1:1000, Abcam, Cambridge, UK, Cat# ab280081), rabbit anti-NSUN6 (1:2000, Proteintech, Wuhan, China, Cat# 17240-1-AP, RRID: AB_2878367), rabbit anti-YTHDC1 (1:500, Proteintech, Cat# 14392-1-AP, RRID: AB_2878052), and rabbit anti-glyceraldehyde-3-phosphate dehydrogenase (1:10,000, Abcam, Cat# ab181602, RRID: AB_2630358; serve as a loading control) overnight at 4°C to detect the proteins of interest in the neuronal samples. The next day, the membranes were incubated with goat anti-rabbit IgG-HRP (1:10,000, Abcam, Cat# ab97080, RRID: AB_10679808) at room temperature for 60 minutes, followed by washing to remove any unbound secondary antibodies. The protein bands were detected using enhanced chemiluminescence detection reagents (Advansta, San Jose, CA, USA). The protein bands were detected using an electrochemiluminescence reagent (Advansta) and a chemiluminescence detector (FluorChem M system, ProteinSimple, San Jose, CA, USA) and quantified using ImageJ 1.54f software (National Institutes of Health, Bethesda, MD, USA). All experiments were repeated three times.

### Cell viability assessment

Cortical neurons were plated in 96-well plates at a density of 1 × 10^4^ cells per well and incubated at 37°C for 7–9 days. After subjecting the cells to a series of treatments, 10 µL of Cell Counting Kit-8 solution (CCK-8; Beyotime, Shanghai, China) was added to each well, and the plates were reincubated for 2 hours. The absorbance at a wavelength of 450 nm was then measured using a multifunctional microplate reader (SpectraMax®i3x, Molecular Devices, San Jose, CA, USA). For the lactate dehydrogenase analysis, a lactate dehydrogenase Cytotoxicity Assay Kit (Beyotime) was used. After subjecting the cells to various treatments, 120 µL of the supernatant was combined with 60 µL of lactate dehydrogenase detection buffer, incubated at room temperature for 30 minutes, and subsequently analyzed at 490 nm using a microplate reader (SpectraMax®i3x).

### Immunofluorescence staining 

Cortical neurons were treated with 0.1% Triton X-100 (Thermo Fisher Scientific, Cat# T8787) at 37°C for 15 minutes to facilitate permeabilization. Then, they were incubated in blocking buffer (TBS containing 0.1% Triton X-100 (Thermo Fisher Scientific, Cat# T8787) and 5% bovine serum albumin) at 37°C for 1 hour. Next, the cells were incubated with a chicken anti-beta III tubulin primary antibody (1:1000, Abcam, Cat# ab41489, RRID: AB_727049) diluted in TBS containing 0.1% Triton X-100 (TBST; Thermo Fisher Scientific, Cat# T8787) and 5% bovine serum albumin overnight at 4°C. The next day, the neurons were washed three times with TBST and incubated with a goat anti-chicken secondary antibody conjugated to Alexa Fluor 594 (1:500, Abcam, Cat# 150172, RRID: AB_10012372) for 2 hours at room temperature. Following three additional washes with TBST, the cultured neurons were stained with the nuclear dye 6-diamidino-2-phenylindole dihydrochloride (DAPI) (Servicebio, Wuhan, China, Cat# G1012) at room temperature for 10 minutes. After performing three final washes with TBST, the cells were imaged by confocal microscopy (LSM 750, Zeiss, Oberkochen, Gottingen, Germany) and quantified using ImageJ 1.54f software.

### Identification of methylation-associated long non-coding RNAs

Weighted gene coexpression network analysis (WGCNA) was performed to identify relationships between lncRNA modules and RNA methylation scores, using a WGCNA package (http://labs.genetics.ucla.edu/horvath/CoexpressionNetwork/) (Langfelder and Horvath, 2008). We discarded outlier samples before selecting lncRNA expression datasets to be used as input. This matrix facilitated the identification of intrinsic co-expression patterns. A soft-thresholding exponent was determined to construct a scale-free network. Following modification of the weighted adjacency matrix, it was converted into a topological overlap matrix. We used a dynamic tree-cutting technique on the topological overlap matrix to cluster lncRNAs hierarchically, attributing a distinct color to each group. This technique led to the identification of a specific lncRNA cluster that demonstrated notable correlation with RNA methylation, warranting further investigation.

### Recognition of molecular subtypes

The brown lncRNA module, in conjunction with datasets GSE48350 and GSE5281, pinpointed specific methylation-associated long non-coding RNAs (MALRs) as being potentially associated with AD. To categorize AD variants within the GSE48350 cohort, a consensus clustering strategy leveraging lncRNA expression profiles was employed. The k-means algorithm, operationalized via the ConsensusClusterPlus (https://bioconductor.org/packages/ConsensusClusterPlus/) package (Wilkerson and Hayes, 2010), was tuned with several predefined parameters: each iteration resampled 80%, the cluster range was set to 2 to 6, and 1000 iterations were executed, all using Euclidean distance metrics. To determine the optimal number of AD molecular subtypes, a variety of metrics were utilized. These included a consensus matrix, a cumulative distribution function (CDF) curve, variations in the area under the CDF curve, and the stability of the cluster allocations. The robustness of the generated clustering outcomes was validated visually by t-SNE.

### Enrichment analysis

Gene ontology (GO) biological function enrichment analysis was performed using the clusterProfiler package (https://github.com/YuLab-SMU/clusterProfiler), as described previously (Yu et al., 2012). Gene set variation analysis (GSVA, https://bioconductor.org/), which is frequently employed to explore biological functions and pathway activities (Hänzelmann et al., 2013), was used to estimate molecular attributes. The distinct molecular features of RNA methylation subtypes were identified using the “c2.cp.kegg.v7.4.symbols” and “c5.go.bp.v7.5.1.symbols” gene sets from the Molecular Signatures Database (MSigDB) (https://www.gsea-msigdb.org/gsea/msigdb/index.jsp). Discrepancies in biological functions and critical pathway alterations were identified using the limma package. GSVA scores with absolute *t*-values greater than 5 were deemed statistically significant.

To identify variations in pathway activities, gene set enrichment analysis (GSEA) analysis was performed using the clusterProfiler package. The Normalized Enrichment Score (NES) was calculated, with an adjusted *P*-value below 0.05 indicating statistical significance. Robust clustering scores were used to validate the integrity of the clustering outcomes, and t-SNE was used to visually verify the cluster structures.

### Immune microenvironment assessment

The immune microenvironment, including immune cell abundance and characteristics, was evaluated using ssGSEA (https://www.genepattern.org/), the Microenvironment Cell Populations counter (MCPcounter; https://github.com/ebecht/MCPcounter), the gene signature deconvolution method (xCell; https://xcell.ucsf.edu/), Absolute Immune Signal (ABIS; https://github.com/giannimonaco/ABIS) deconvolution, and ESTIMATE (https://cibersort.stanford.edu/). These methods were used to compute enrichment scores for, and the relative proportions of, each immune cell subtype. The Wilcoxon rank-sum test was used to compare immune cell proportions across different groups. Furthermore, the ESTIMATE package was used to compute the immune score and analyze the expression of genes involved in immunomodulation, thus exploring variations in immune potential.

### Selection of characteristic lncRNAs

Characteristic RNA methylation-associated lncRNAs were identified using learning algorithms, including least absolute shrinkage and selection operator (LASSO), Random Forest (RF), and XGBoost models, based on the glmnet (https://github.com/cran/glmnet), caret (http://www.nitrc.org/projects/caret/), Boruta (https://CRAN.R-project.org/package=Boruta), and xgboost (https://github.com/dmlc/xgboost) packages. LASSO involves the estimation of tuning parameters that control the number of constraints, optimized by minimizing the cross-validation mean squared error of the predictions (Bøvelstad et al., 2007; Hu et al., 2017). The coefficients of variables were determined according to the optimal penalty parameter λ based on at least 10-fold cross-validation (Engebretsen and Bohlin, 2019). To identify important lncRNAs, the Boruta algorithm was used with the following parameters: max iterations equal to 300, *P*-value less than 0.01. Following the completion of 300 iterations, lncRNAs whose classification remained uncertain and those rejected by the algorithm were excluded from further analysis. The caret package was subsequently utilized to fit the significant lncRNAs identified by the Boruta algorithm to a random forest model with default parameters. The importance of these lncRNAs was then ranked on the basis of calculated values. The XGBoost classifier, a distributed gradient boosting algorithm, was used to transform weak variables into strong ones (Chen et al., 2015) by implementing the xgboost package with default parameter values. Weights were applied to calculate the MALRs, validating their pertinence to the XGBoost model. The 10 lncRNAs with the greatest weights were identified as being related to the XGBoost model, and Shapley Additive exPlanation (SHAP) values were used to further elucidate the model. To mitigate over-fitting, a 10-fold cross-validation approach was adopted across all three machine learning models. The samples in the GSE48350 dataset were randomly divided into a training set comprising 70% of the data and a test set containing 30%. lncRNAs characterized as significant by all three machine learning models were ultimately identified. Two independent datasets—GSE5281 and GSE28146—were selected as external validation cohorts, and the area under the curve (AUC) was utilized to evaluate diagnostic performance using the pROC package (https://www.r-project.org/).

### Construction of a nomogram and risk model related to adverse reactions

To develop a predictive nomogram, the identified MALRs were applied using the “rms” package (https://CRAN.R-project.org/package=rms). The ESTIMATE package was employed to calculate the immune score and investigate the expression of genes associated with immunomodulation, thereby examining differences in immune potential. Additionally, decision curve analysis (DCA) results derived from the ggDCA package (https://www.r-project.org/) were used to measure and appraise the clinical advantages of the nomogram. MALR risk scores were determined using coefficients derived from the final lncRNA characteristics pinpointed by the LASSO model: risk score = Σ (Coefficientsi × lncRNA expression level). The five characteristic MALRs with corresponding coefficients in the LASSO model were employed to calculate the RNA methylation-lncRNA risk score, as follows: riskScore = (–0.191284 × LINC01007) + (–0.007843 × MAP4K3-DT) + (0.121186 × MIR302CHG) + (0.205274 × VAC14-AS1) + (0.402381 × TGFB2-OT1). Subsequently, the capability of the riskScore and individual characteristic MALRs to forecast AD progression in the GSE48350 dataset was assessed. The AD sample set was sorted according to these risk scores. The median score served as a cutoff, classifying samples as either low-risk (below the median) or high-risk (above it). Leveraging these stratified MALRs, a risk assessment framework was constructed, and 80 AD subjects from GSE48350 were classified into the respective risk categories. The robustness of this risk score system was later affirmed in two distinct datasets: GSE5281 and GSE28146.

### Identification of candidate therapeutic drugs

Connectivity Map (CMap) is a versatile tool used to identify potential therapeutic agents for patients at varying risk levels by analyzing their gene expression profiles (Yang et al., 2022). Drug profiles were obtained from the CMap database (https://clue.io/). The gene expression profiles of the top 100 most overexpressed and underexpressed genes in high-risk and low-risk patients were evaluated. Alignment between drug signatures and gene expression patterns was examined. CMap scores were computed utilizing the eXtreme Sum (XSum) method, following protocols described earlier (Cheng et al., 2014; Yang et al., 2022).

### Statistical analysis

Statistical analyses and data visualization were performed using R version 4.1.0 (https://cran.r-project.org/). To investigate the relationship between two continuous variables, Spearman’s rank correlation method was applied. Comparison of continuous data between two distinct groups was carried out using the Wilcoxon rank-sum test and the unpaired *t*-test. Receiver Operating Characteristic (ROC) curve analysis was employed to evaluate the predictive power concerning binary outcomes. Additionally, to adjust *P*-values, the Benjamini–Hochberg procedure was implemented, setting the significance threshold at *P*-values less than 0.05.

## Results

### Dysregulation of RNA methylation regulators may contribute to Alzheimer’s disease development

**Figure 1:**
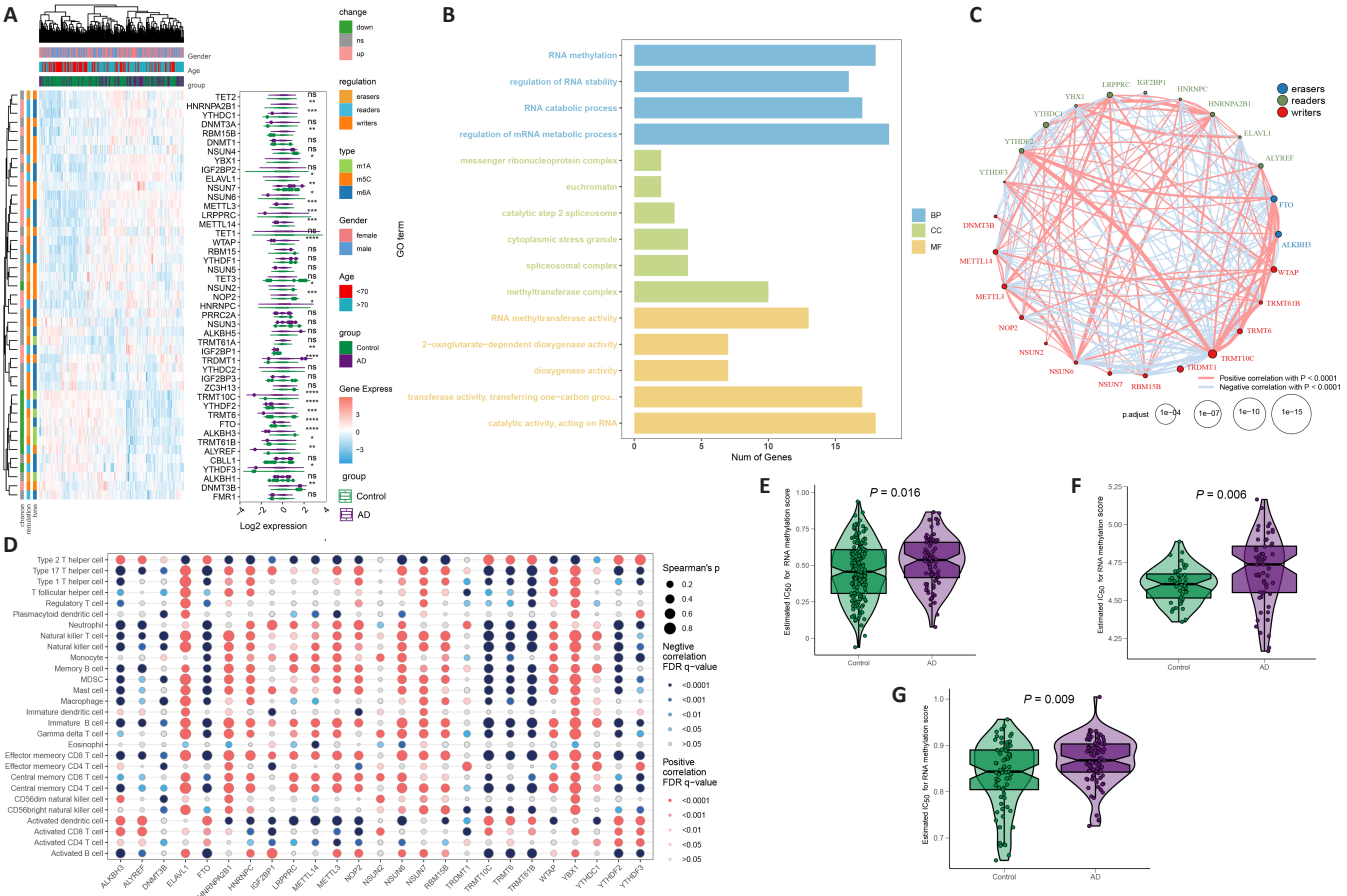
Evaluation of differences in RNA methylation modifications between patients with AD and healthy controls. (A) Heatmap (left) and violin plot (right) of the expression levels of 45 RNA methylation regulators in AD and non-AD brain tissues from GSE48350, annotated by age, sex, Mini-Mental State Examination (MMSE), and Braak stage. Horizontal lines represent median and interquartile range. *P < 0.05, **P < 0.01, ***P < 0.001, ****P < 0.0001, *vs*. control group (Wilcoxon rank sum test). ns: not significant. (B) Barplots exhibiting GO enrichment analysis of 45 RNA methylation regulators. (C) Interactions among differentially expressed RNA methylation regulators. The circle size represents the effect of individual variables on AD. The Benjamini–Hochberg approach was used to adjust the *P*-values. The different colors represent the distinct functions of the RNA methylation regulators. Each line connecting DEGs represents an interaction, and the thickness of each line corresponded to the strength of the correlation. Red indicates a positive correlation, and blue indicates a negative correlation. (D) Correlations between differentially expressed RNA methylation regulators and 28 immune cell subtypes. The circle size represented the strength of the correlation. Red indicates positive correlations, and blue indicates negative correlations. The shade of the color indicates the adjusted *P*-value. (E–G) Comparison of RNA methylation score between AD and non-AD brain (control) tissues in GSE48350 (E), GSE122063 (F), and GSE5281 (G). Horizontal lines indicate median and interquartile range. AD: Alzheimer’s disease; DEGs: differential expressed genes; GO: Gene Ontology.

To elucidate the association between RNA methylation and AD, we initially obtained the expression profiles of 45 RNA methylation regulation genes from the GSE48350 dataset. These genes comprise 25 writers, 13 readers, and 7 erasers. Abnormal RNA methylation regulators expression patterns were apparent in the brain tissues of patients with AD compared with healthy controls, indicating distinct biological functions between patients with AD and healthy individuals. The heatmap and violin plot illustrated the expression profiles of RNA methylation regulators in patients with AD (**Figure 1A**). In AD brain tissue, the expression levels of 25 of the RNA methylation regulators (13 writers, 10 readers, and 2 erasers) were statistically significant from those seen in healthy controls. Among them, insulin-like growth factor 2 mRNA-binding protein 1 (IGF2BP1), tRNA aspartic acid methyltransferase 1, RNA binding motif protein 15B, heterogeneous nuclear ribonucleoprotein A2B1 (HNRNPA2B1), YTHDC1, Y-box binding protein 1 (YBX1), embryonic lethal-abnormal vision like protein 1, NSUN7, NSUN6, methyltransferase-like protein 3 (METTL3), leucine-rich PPR motif-containing protein, METTL4, WT1-associated protein, NOP2, heterogeneous nuclear ribonucleoprotein C (HNRNPC), and DNMT3B were overexpressed in AD tissues relative to normal brain tissues. GO annotation suggested that these RNA methylation regulators were mainly enriched in RNA modification and methylation, regulation of RNA stability, RNA catabolic and metabolic processes, regulation of the spliceosome and the methyltransferase complex, and other processes (**Figure 1B**). Subsequently, control and AD samples were divided by patient age, and changes in age-related RNA methylation regulators were assessed in detail (**Additional Figure 1**). We found that AlkB homolog 3, tRNA methyltransferase 6, FTO, tRNA methyltransferase 10C, and YTHDF2 were significantly expressed at all ages in the control group, while increased expression of YBX1, embryonic lethal-abnormal vision like protein 1, HNRNPA2B1, NSUN6, and IGF2BP1 were observed in patients with AD aged less than 75. Furthermore, in the AD group, the expression of NSUN2, RNA binding motif protein 15B, NSUN7, and heterogeneous nuclear ribonucleoprotein C gradually increased with age, peaked around 61–75 years, and eventually decreased (**Additional Figure 1**). The interactions among these regulators, such as synergistic effects between YBX1 and HNRNPA2B1 and antagonistic effects between TRMR10C and NSUN6, suggest that they play a pivotal role in AD progression (**Figure 1C**). First, we identified differences in immune cell subsets between control subjects and patients with AD. Activated B cells, central memory CD4 T cells, effector memory CD4 T cells, effector memory CD8 T cells, immature B cells, immature dendritic cells, mast cells, myeloid-derived suppressor cells, memory B cells, natural killer cells, natural killer T cells, neutrophils, regulatory T cells, type 1 T helper cells, and type 17 T helper cells were enriched in patients with AD, suggesting that a dysregulated immune microenvironment plays a critical role in AD pathology (**Additional Figure 2**). Next, we analyzed correlations among 25 differentially expressed RNA methylation regulators and 28 immune cell subtypes (**Figure 1D**). The results indicated that the expression levels of these RNA methylation regulators correlated markedly with immune cell counts, suggesting the crucial role of RNA methylation in the AD immune microenvironment. Next, the RNA methylation score was determined via ssGSEA to gain deeper insight into the interaction between RNA methylation and AD. Patients with AD in the GSE48350 dataset had higher RNA methylation scores (*P* = 0.016, Cohen’s *d* = 0.343; **Figure 1E**), which was consistent with the results from two independent datasets: GSE122063 (*P* = 0.006, Cohen’s *d* = 0.507) and GSE5281 (*P* = 0.009, Cohen’s *d* = 0.460; **Figure 1F** and **G**). Taken together, these results suggest that RNA methylation regulators are strongly connected to AD and might have a substantial impact on AD progression.

### Heterogeneity of RNA methylation levels across cell types in patients with Alzheimer’s disease

**Figure 2:**
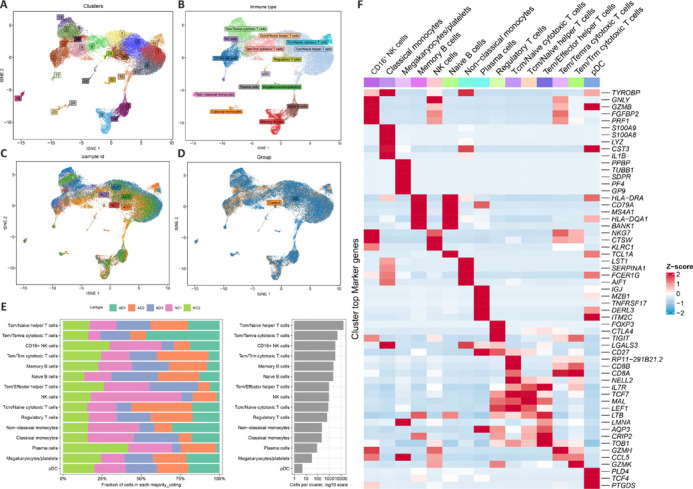
Cell subtype analysis based on single-cell gene expression. (A–D) Cell clusters (A), cell type annotations (B), sample (C), and group (D) distribution based on the uniform manifold approximation and projection (UMAP) plot of 36,717 cells. (E) Cell type fraction of each sample in GSE181279. (F) Representative heatmap showing the expression profiles of the top five unique signature genes in each cell annotation cluster.

To further understand the link between RNA methylation and AD, we analyzed a scRNA-seq dataset comprising blood samples from two healthy individuals and three patients with AD. Post-quality control, 16,727 distinct genes across 36,717 cells from all five samples were retained. After gene expression normalization, principal component analysis and uniform manifold approximation and projection (UMAP)-based clustering were conducted within a principal component analysis space comprising 30 principal components. This analysis delineated 25 discrete cell clusters characterized by DEGs (**Figure 2A**). The “Celltypist” algorithm was utilized to classify these clusters into known cell subtypes, including various T cell populations (Tem/effector helper T, Tem/Temra cytotoxic T, Tem/Trm cytotoxic T, Tcm/naive cytotoxic T, Tcm/naive helper T, and regulatory T cells), B cells (memory and naive), NK cells, CD16^+^ NK cells, monocytes (non-classical and classical), plasmacytoid dendritic cells, plasma cells, and megakaryocytes/platelets (**Figure 2B**). Moreover, UMAP visualization revealed the cellular distribution according to the origin and category of each sample (**Figure 2C** and **D**). The proportions of cell types per sample are depicted in **Figure 2E**, highlighting an enrichment of certain cell types such as Tcm/naive helper T, Tem/Temra and Tem/Trm cytotoxic T cells, naive B cells, Tcm/naive cytotoxic T cells, regulatory T cells, megakaryocytes/platelets, and plasmacytoid dendritic cells in the AD samples, whereas CD16^+^ NK cells, NK cells, and plasma cells were more prevalent in the healthy controls. The expression patterns of the leading five signature genes for each cell type, illustrated in **Figure 2F**, showcase the ability of these genetic markers to effectively distinguish among distinct subtypes.

**Figure 3:**
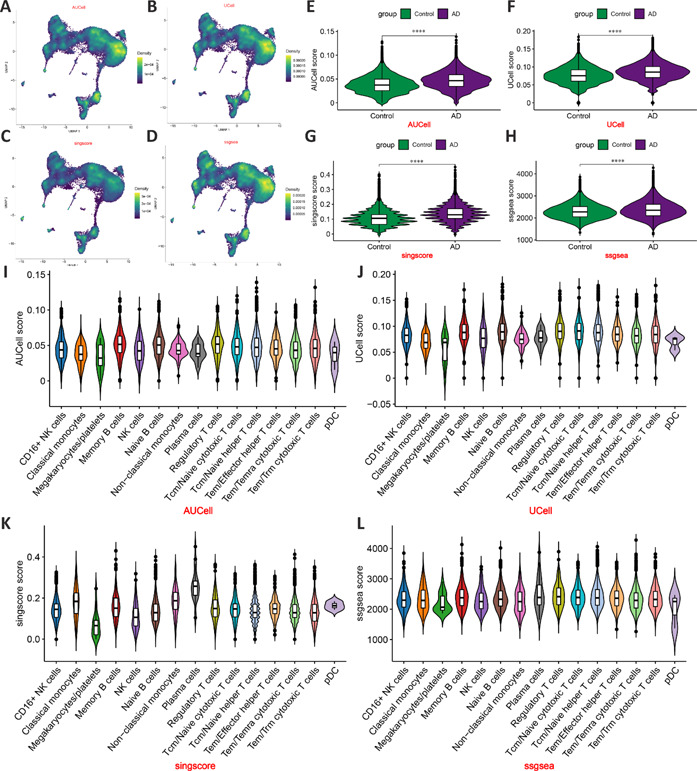
Evaluation of RNA methylation scores at the single-cell level. (A–D) Distribution of the RNA methylation scores for each cell based on the AUCell (A), UCell (B), singscore (C), and ssgsea (D) algorithms, visualized as UMAP plots. (E–H) Representative violin plots showing differences in the RNA methylation score based on the AUCell (E), UCell (F), singscore (G), and ssgsea (H) algorithms between normal and AD samples. Horizontal lines indicate median and interquartile range. ****P < 0.0001 (Wilcoxon rank sum test). (I–L) Representative violin plots showing differences in the RNA methylation scores based on the AUCell (I), UCell (J), singscore (K), and ssgsea (L) algorithms among infiltrating immune cells in AD. AD: Alzheimer’s disease; UMAP: Uniform manifold approximation and projection.

Next, various scoring algorithms were used to quantify RNA methylation scores at the single-cell level. All algorithms demonstrated that patients with AD had significantly higher RNA methylation scores than those in the normal group, which is consistent with the results from analysis of the bulk transcriptome data (**Figure 3A–H**). The scRNA-seq data from infiltrating immune cells in AD were further extracted and analyzed. Interestingly, the AUCell, UCell, and ssgsea algorithms yielded high RNA methylation scores for T cells, B cells, and NK cells compared with other cell types. By contrast, the singscore approach showed the most significant scores for plasma cells and monocytes (**Figure 3I–L**). To further explore the mechanism by which RNA methylation effectors contribute to AD pathology, we specifically analyzed the expression of RNA methylation effectors in different cell types derived from brain tissues. Among 103,988 high-quality cells, we identified 19 clusters based on unsupervised clustering and UMAP reduction. These clusters were annotated as eight cell types (astrocytes, microglia, oligodendrocytes, excitatory neurons, inhibitory neurons, oligodendrocyte progenitor cells, endothelial cells, and fibroblasts), based on various well-established marker genes. The ssgsea algorithm showed that RNA methylation scores were highly expressed in astrocytes, microglia, and oligodendrocyte progenitor cells (**Additional Figure 3**). These findings indicate elevated RNA methylation levels may contribute to AD progression.

### Dysregulation of crucial RNA methylation regulators in an *in vitro* model of Alzheimer’s disease

**Figure 4:**
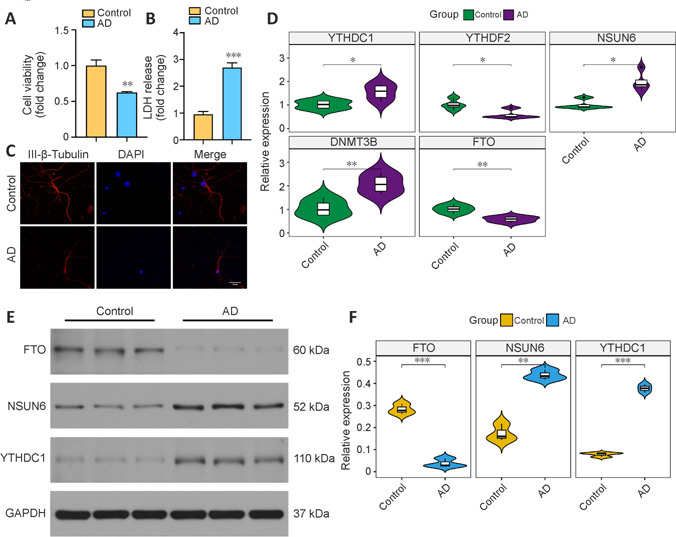
Validation of RNA methylation regulators *in vitro*. (A) Cell viability was detected by Cell Counting Kit-8 assay. (B) Cell cytotoxicity was determined by LDH release assay. For A and B, data are expressed as mean ± SEM (*n* = 3). (C) Localization of immunoreactive class III-β-tubulin on neurons before and after Aβ oligomer-induced damage. The left column displays the neuronal marker class III-β-tubulin (red) and nuclei stained with DAPI (blue). The right column shows co-localization of class III-β-Tubulin and DAPI. Scale bar: 20 μm. (D) Representative violin plots showing differences in the expression of YTHDC1, YTHDF2, NSUN6, DNMT3B, and FTO; horizontal lines indicate median and interquartile range (*n* = 4). (E) Representative immunoblots and (F) western blot analysis of FTO, NSUN6, and YTHDC1. For D and F, horizontal lines indicate median and interquartile range (*n* = 3). *P < 0.05, **P < 0.01, ***P < 0.001, *vs*. control group (unpaired *t*-test). DNMT3B: DNA-methyltransferase-3B; FTO: Fat mass and obesity-associated; LDH: lactate dehydrogenase; NSUN6: NOP2/Sun RNA methyltransferase 6; YTHDC1: YTH domain-containing protein 1; YTHDF2: YTH domain family 2.

To validate the expression of RNA methylation regulators in AD, an *in vitro* AD model was constructed by treating primary cortical neurons with Aβ oligomers. It was found that Aβ oligomers resulted in reduced cell viability, increased toxicity, and neuronal synaptic damage (**Figure 4A–C**). One eraser (FTO), two readers (YTHDC1 and YTHDF2), and two writers (NSUN6 and DNMT3B) were selected for subsequent qRT-PCR analysis. We found significantly higher expression levels of YTHDC1, NSUN6, and DNMT3B and significant downregulation of FTO and YTHDF2 in AD-treated cortical neurons than in normal neurons (**Figure 4D**). In alignment with the qRT-PCR findings, FTO expression was reduced, whereas NSUN6 and YTHDC1 expression levels were increased, following AD injury (**Figure 4E** and **F**). These results validate that RNA methylation regulator expression levels are dysregulated in AD.

### RNA methylation–associated long non-coding RNAs may contribute to Alzheimer’s disease pathogenesis

**Figure 5:**
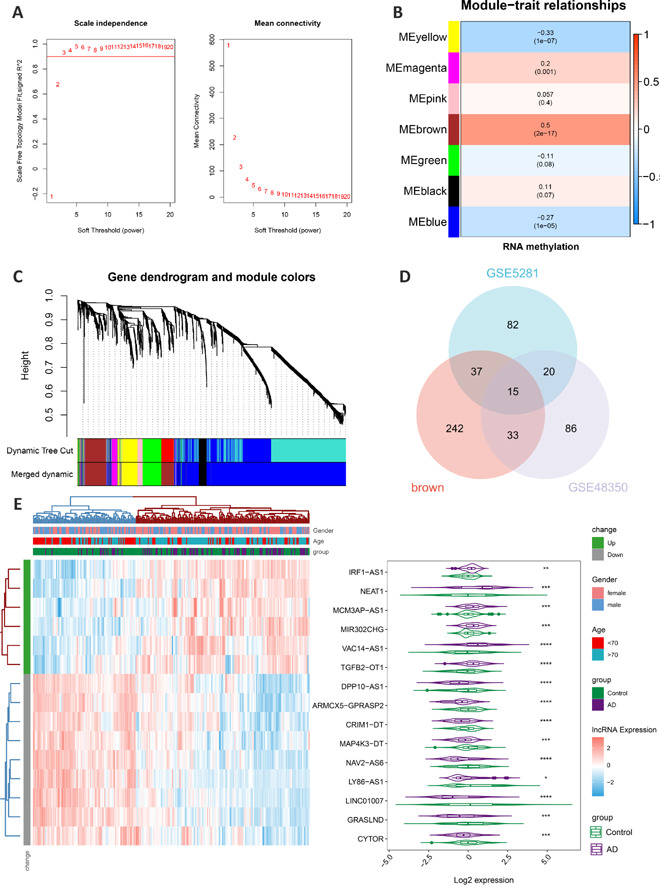
Identification of MALRs. (A) Selection of soft threshold β. (B) Clustering dendrograms of co-expression modules; each color corresponds to an individual module. (C) Heatmap showing correlations between the seven modules and RNA methylation score. (D) Venn diagram displaying common MALRs among the brown module, the GSE48350 DElncRNAs, and the GSE5281 DElncRNAs. (E) Heatmap (left) and violin plot (right) of the expression profiles of 15 MALRs between AD and non-AD brain tissues in GSE48350, annotated by age, sex, MMSE, and Braak stage. Horizontal lines represent median and interquartile range. *P < 0.05, **P < 0.01, ***P < 0.001, ****P < 0.0001, *vs*. control group (Wilcoxon rank sum test). AD: Alzheimer’s disease; MALRs: methylation-associated lncRNAs; MMSE: Mini-Mental State Examination.

To further elucidate RNA methylation regulation patterns, we performed WGCNA to identify MALRs. A network exhibiting scale-free topology and connectivity was generated using the PickSoftThreshold function by setting the soft threshold β to 3, resulting in a no-scale *R*^2^ value approaching 0.9 (**Figure 5A**). Then, a hierarchical clustering algorithm was applied to the cluster dendrogram, generating seven differently colored lncRNA co-expression modules. The brown module exhibited the strongest correlation with the RNA methylation score (*R* = 0.5; **Figure 5B** and **C**). Therefore, the lncRNAs inside the brown module were used in subsequent analyses. We identified 15 MALRs that were common among the brown module lncRNAs, GSE48350 DElncRNAs, and GSE5281 DElncRNAs (**Figure 5D**). The distinct expression patterns of these 15 MALRs between patients with AD and controls are shown in **Figure 5E**. Specifically, the expression levels of IRF1-AS1, NEAT1, MCM3AP-AS1, MIR302CHG, VAC14-AS1, and TGFB2-OT1 were markedly elevated in patients with AD compared with controls. Conversely, DPP10-AS1, ARMCX5-GPRASP2, CRIM1-DT, MAP4K3-DT, NAV2-AS6, LY86-AS1, LINC01007, GRASLND, and CYTOR were significantly downregulated in patients with AD compared with controls. These findings suggest a possible association between these lncRNAs and AD pathogenesis.

### Identification of two distinct MALR subtypes in patients with Alzheimer’s disease

**Figure 6:**
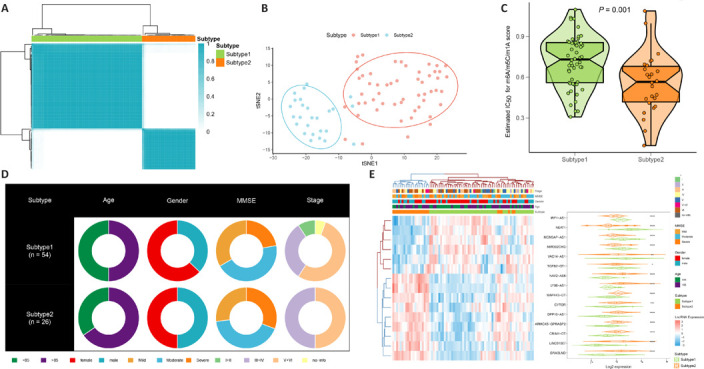
Identification of RNA methylation subtypes by consensus clustering of data from patients with AD. (A) RNA methylation subtype matrix constructed using a consensus clustering approach. (B) t-SNE diagram showing clustering of the subtype 1 and subtype 2 samples. (C) Comparison of RNA methylation scores between subtype 1 and subtype 2 in patients with AD. Horizontal lines indicate median and interquartile range (Wilcoxon rank sum test). (D) Pie charts showing the differences in clinical features between subtype 1 and subtype 2 patients with AD. (E) Heatmap (left) and violin plot (right) of the expression profiles of 15 MALRs between subtype 1 and subtype 2 in patients with AD, annotated by age, sex, MMSE, and Braak stage. AD: Alzheimer’s disease; MALRs: methylation-associated lncRNAs; MMSE: Mini-Mental State Examination; t-SNE: t-Distributed Stochastic Neighbor Embedding.

Different N6-methyladenosine modification patterns may affect diseases in various ways. To further explore the potential roles of RNA methylation in AD, we performed consensus clustering of the expression levels of the 15 MALRs to classify patients with AD into different subtypes. Each subtypes represents a unique state of RNA methylation modification (**Additional Figure 4**). A higher consensus matrix score indicated a greater likelihood of samples being grouped, as depicted in **Figure 6A**. Additionally, a smoother central portion of the CDF curve corresponded to more precise sample classification. By merging these findings with the evaluation of modifications in the area beneath the CDF curve and uniform cluster scores, we definitively identified two patient subtypes. tSNE analysis revealed notable differences between subtype 1 and subtype 2 (**Figure 6B**). In addition, RNA methylation subtype 1 was associated with a higher RNA methylation score (*P* = 0.001, *Cohen’s d* = 0.802; **Figure 6C**), suggesting a more significant RNA methylation modification pattern in subtype 1. **Figure 6D** presents the differences in clinical features, including age, sex, Mini-Mental State Examination (MMSE), and stage, between subtype 1 and subtype 2. No notable differences in age, sex, or MMSE were observed between the two subtypes (*P* = 0.24, 0.33, and 0.72, respectively), whereas marked differences in the expression patterns of the 15 MALRs were also observed in subtype 1 relative to subtype 2 (**Figure 6E**). To validate our RNA methylation subtypes, we assessed methylation scores in the GSE5281 dataset and, in line with the results from the GSE48350 dataset, observed relatively high RNA methylation scores for RNA methylation subtype 1. The two RNA methylation subtypes were also clearly present in the GSE5281 dataset (**Additional Figure 5**). These results imply that RNA methylation exhibits distinct modification states in AD.

### Distinct molecular functions and pathways between RNA methylation subtypes

**Figure 7:**
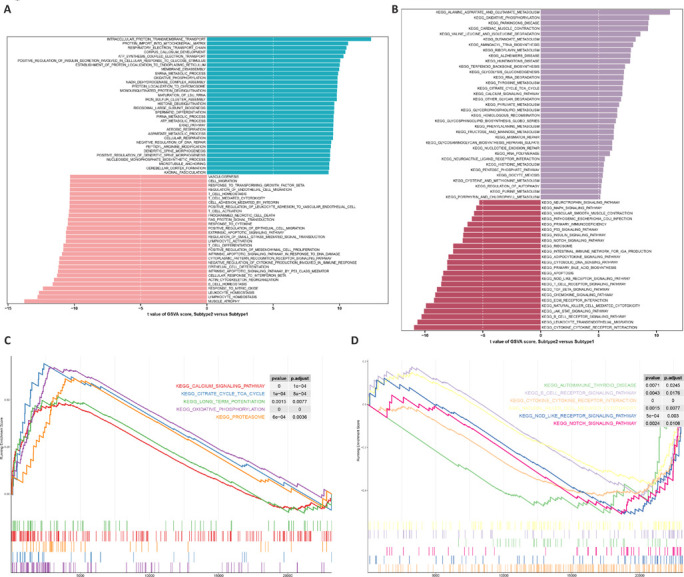
Functional enrichment analysis of the RNA methylation subtypes. (A, B) Differences in abundant biological functions (A) and characteristic signaling pathways (B) between subtype 1 and subtype 2, ranked by the *t*-value of the GSVA scores. (C, D) GSEA showing upregulated (C) and downregulated (D) key pathways in subtype 2 patients. GSEA: Gene set enrichment analysis; GSVA: gene set variation analysis.

Understanding the roles and potential mechanisms of the distinct RNA methylation subtypes in AD is essential for uncovering their therapeutic potential. Therefore, variances in the transcriptome profiles of these RNA methylation subtypes were elucidated through DEG analysis. In total, 3511 DEGs were identified between subtype 1 and subtype 2, including 1399 upregulated genes and 2112 downregulated genes (**Additional Figure 6**). Functional annotation based on GSVA indicated that the functions of the DEGs in RNA methylation subtype 1 were mainly enriched in cell migration and differentiation, cell death regulation, and the immune response, including T cell and B cell homeostasis and lymphocyte activation, whereas those in RNA methylation subtype 2 were closely related to energy metabolism–related biological functions, including oxidative phosphorylation, the respiratory electron transport chain, the ATP metabolic process, and cellular respiration (**Figure 7A**). Pathway enrichment analysis indicated that the RNA methylation subtype 1 was predominantly driven by pathways related to immunity, apoptosis, chemokines, and cytokine interaction, with immune regulation being the most enriched pathway. Subtype 2 notably regulated pathways associated with neurodegenerative diseases and metabolism (**Figure 7B**). Consistent with this, the GSEA results showed that subtype 2 was characterized by elevated calcium signaling pathway, TCA cycle, long-term potentiation, oxidative phosphorylation, and proteasome activities (**Figure 7C**). Subtype 2 was also involved in autoimmune disease, the B cell receptor signaling pathway, cytokine and cytokine receptor interactions, natural killer cell–mediated cytotoxicity, the NOD-like receptor, and the Notch signaling pathway (**Figure 7D**). The results reveal the specific roles of these two RNA methylation subtypes in AD progression.

### Distinct immune and metabolic characteristics of the two RNA methylation subtypes

**Figure 8:**
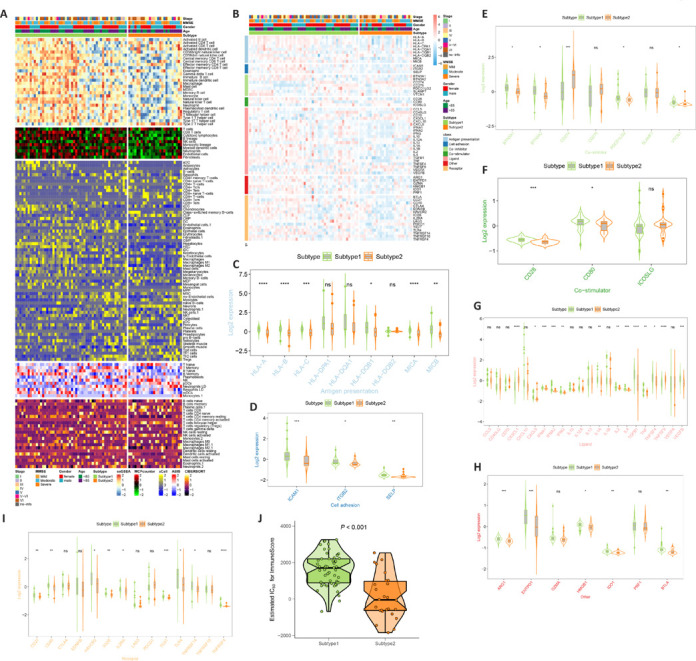
Immune characteristics of the different RNA methylation subtypes. (A) Heatmap of the expression profiles of infiltrated immune cells based on the ssGSEA, MCPcounter, xCell, ABIS, and ESTIMATE algorithms between subtypes 1 and 2 in patients with AD. (B) Heatmap showing the expression profiles of immunoregulatory subgroup genes between subtypes 1 and 2 in patients with AD, annotated by age, sex, MMSE, and Braak stage. (C–I) Violin plots exhibiting differences in the expression of immunoregulatory genes linked to antigen presentation (C), co-stimulator (D), cell adhesion (E), co-inhibitor (F), ligand (G), receptor (H), and other molecules (I) between subtypes 1 and 2 in patients with AD. (J) Comparison of immune scores between subtypes 1 and 2 in patients with AD. Horizontal lines represent median and interquartile range. *P < 0.05, **P < 0.01, ***P < 0.001, ****P < 0.0001, *vs*. subtype 1 group (Wilcoxon rank sum test). ABIS: Amputee Body Image Scale; AD: Alzheimer’s disease; MCPcounter: Microenvironment Cell Populations counter; MMSE: Mini-Mental State Examination; ns: not significant; ssGSEA: single sample gene set enrichment analysis; xCell: gene signature deconvolution method.

To elucidate differences in the immunological properties of these subtypes, we comprehensively assessed immune cell subset infiltration levels using ssGSEA, MCPcounter, xCell, ABIS, and ESTIMATE. As illustrated in **Figure 8A** and **Additional Figure 7**, most immune cells, such as CD4^+^ T cells, CD8^+^ T cells, NK T cells, neutrophils, and dendritic cells, were found in RNA methylation subtype 1. Subsequently, we compared immune regulatory gene expression between the subtypes (**Figure 8B**). Notably, RNA methylation subtype 1 was characterized by an immune-active phenotype, while subtype 2 was characterized by a metabolic phenotype. The immune-active subtype exhibited higher immune infiltration, upregulated immune regulatory gene expression, and elevated immune scores, suggesting a more significant immune response (**Figure 8C–J**). In addition, to understand the immune and metabolic landscape across all brain cell types in AD, we analyzed metabolic pathway activity using scMetabolism. Excitatory neurons and inhibitory neurons exhibited the highest metabolic activity scores, while the metabolic activity of astrocytes and oligodendrocytes was significantly decreased. In addition, microglia exhibited significantly increased scores related to the inflammatory response, followed by the astrocytes, endothelial cells, and fibroblasts (**Additional Figure 8**), revealing that distinct brain cell types have different phenotypes.

### Crucial methylation-associated long non-coding RNAs identified by multiple machine learning algorithms accurately predict Alzheimer’s disease onset

**Figure 9:**
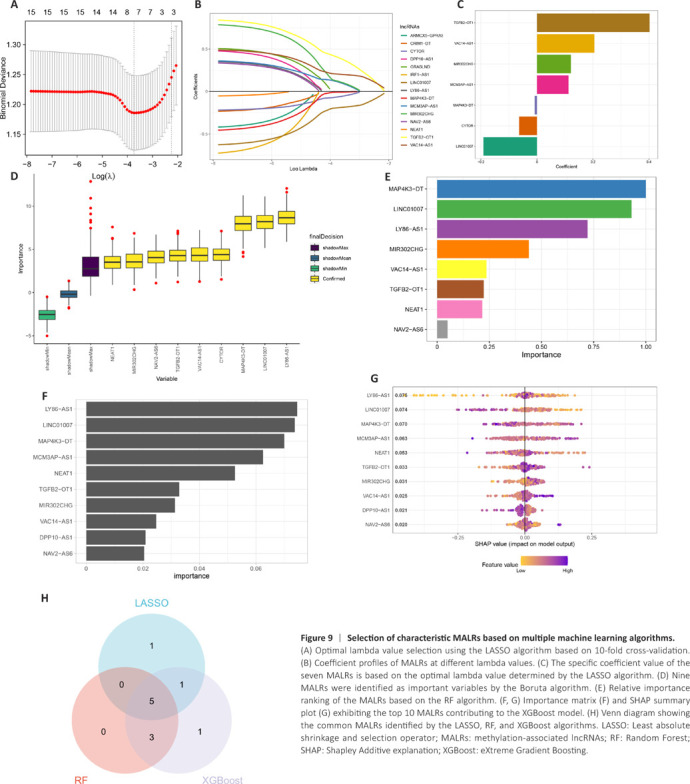
Selection of characteristic MALRs based on multiple machine learning algorithms. (A) Optimal lambda value selection using the LASSO algorithm based on 10-fold cross-validation. (B) Coefficient profiles of MALRs at different lambda values. (C) The specific coefficient value of the seven MALRs is based on the optimal lambda value determined by the LASSO algorithm. (D) Nine MALRs were identified as important variables by the Boruta algorithm. (E) Relative importance ranking of the MALRs based on the RF algorithm. (F, G) Importance matrix (F) and SHAP summary plot (G) exhibiting the top 10 MALRs contributing to the XGBoost model. (H) Venn diagram showing the common MALRs identified by the LASSO, RF, and XGBoost algorithms. LASSO: Least absolute shrinkage and selection operator; MALRs: methylation-associated lncRNAs; RF: Random Forest; SHAP: Shapley Additive explanation; XGBoost: eXtreme Gradient Boosting.

To identify key MALRs that accurately forecast AD progression, we used three machine-learning algorithms to perform feature selection from the expression profiles of 15 MALRs. An optimal lambda value of 0.0238, which was associated with the highest accuracy, was fitted to the LASSO model and identified seven MALRs (TGFB2-OT1, VAC14-AS1, MIR302CHG, MCM3AP-AS1, MAP4K3-DT, CYTOR, and LINC01007) with non-zero coefficients (**Figure 9A–C**). The Boruta algorithm confirmed nine MALRs as being important variables (**Figure 9D**). The characteristic MALRs determined by the RF model based on the Boruta algorithm are ranked by importance in **Figure 9E**. Furthermore, analysis of the SHAP summary plot for the weight ranking of MALR features in the XGBoost model indicated that the top 10 MALRs contributing to the model’s performance included LY86-AS1, LINC01007, MAP4K3-DT, MCM3AP-AS1, NEAT1, TGFB2-OT1, MIR302CHG, VAC14-AS1, DPP10-AS1, and NAV2-AS6 (**Figure 9F**). We then used SHAP-dependent analysis to show how individual MALR characteristics influenced the XGBoost prediction model results (**Figure 9G**). As the SHAP value of a MALR increased, the probability of AD occurrence also increased. In the XGBoost algorithm, decreased LINC01007 feature values were connected to positive SHAP values and exhibited a strong correlation with increased risk of AD. However, as the value of LINC01007 decreased, the corresponding SHAP value increased. More detailed information on the top 10 most important MALRs that influence the results of the XGBoost model is presented in **Additional Figure 9**. Finally, analysis of the intersection among the results from the three algorithms identified five characteristic MALRs (LINC01007, MAP4K3-DT, MIR302CHG, VAC14-AS1, and TGFB2-OT1) (**Figure 9H**) with high diagnostic value in predicting AD onset.

### The risk model constructed based on methylation-associated long non-coding RNAs exhibits clinical utility for predicting Alzheimer’s disease

**Figure 10:**
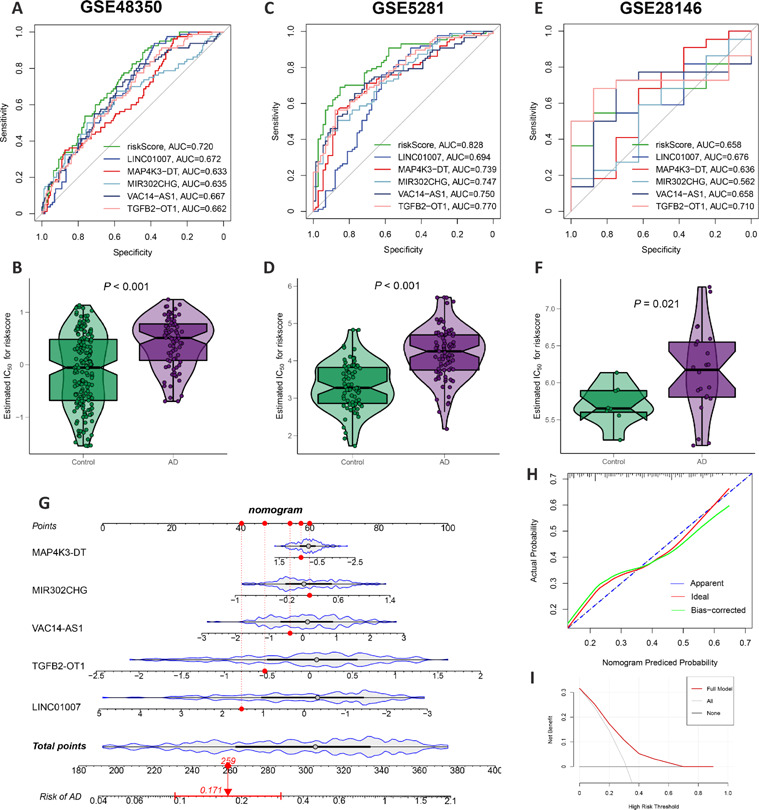
Risk score construction and validation of the diagnostic efficacy of the five characteristic MALRs. (A) ROC curves showing the diagnostic efficacy of the risk score and characteristic MALRs in the GSE48350 dataset. (B) Comparison of risk scores between AD and non-AD brain tissues in the GSE48350 dataset. (C) ROC curves show the diagnostic efficacy of the risk score and characteristic MALRs in the GSE5281 dataset. (D) Comparison of risk scores between AD and non-AD brain tissues in the GSE5281 dataset. (E) ROC curves showing the diagnostic efficacy of the risk score and characteristic MALRs in the GSE28146 dataset. (F) Comparison of risk scores between AD and non-AD brain tissues in the GSE28146 dataset. (G) Predictive nomogram based on the five characteristic MALRs. (H) Calibration curve exhibiting the predicted efficacy of the nomogram. (I) DCA exhibiting the clinical benefits of the nomogram. Horizontal lines indicate median and interquartile range (Wilcoxon rank sum test). AD: Alzheimer’s disease; DCA: decision curve analysis; MALRs: methylation-associated lncRNAs; ROC: Receiver Operating Characteristic.

The AUC values of these curves were identified as 0.720, 0.672, 0.633, 0.635, 0.667, and 0.662 for riskScore, LINC01007, MAP4K3-DT, MIR302CHG, VAC14-AS1, and TGFB2-OT1, respectively. A notable elevation in riskScore was detected in AD samples when compared with normal brain tissue (*P* < 0.001, *Cohen’s d* = 0.882; **Figure 10A** and **B**). Two other independent validation datasets—GSE5281 and GSE28146—were used for external validation. In GSE5281, the AUC values were 0.828, 0.694, 0.739, 0.747, 0.750, and 0.770 for riskScore, LINC01007, MAP4K3-DT, MIR302CHG, VAC14-AS1, and TGFB2-OT1, respectively, and patients with AD exhibited significantly higher riskScores (*P* < 0.001, *Cohen’s* *d* = 1.324; **Figure 10C** and **D**). In GSE28146, the AUC values were 0.658, 0.676, 0.636, 0.562, 0.658, and 0.710 for riskScore, LINC01007, MAP4K3-DT, MIR302CHG, VAC14-AS1, and TGFB2-OT1, respectively. Consistent with these results, patients with AD also showed elevated riskScores in GSE28146 (*P* = 0.021, *Cohen’s* *d* = 0.587; **Figure 10E** and **F**). These results demonstrated that the riskScore enabled more accurate prediction of AD progression. Next, a nomogram including each characteristic MALR was set up to predict the risk of AD. The results indicated that the MALRs were all associated with satisfactory prediction outcomes (**Figure 10G**). The calibration plot confirmed the nomogram’s precision in forecasting AD risk (**Figure 10H**). In the Decision Curve Analysis (DCA), the *y*-axis depicts the net benefits, whereas the *x*-axis indicates the threshold probability, demonstrating the potential clinical benefit of the nomogram (**Figure 10I**). These results reveal that our risk model exhibits clinical utility for predicting AD onset.

### Differences in molecular pathways between the high- and low-risk groups

**Figure 11:**
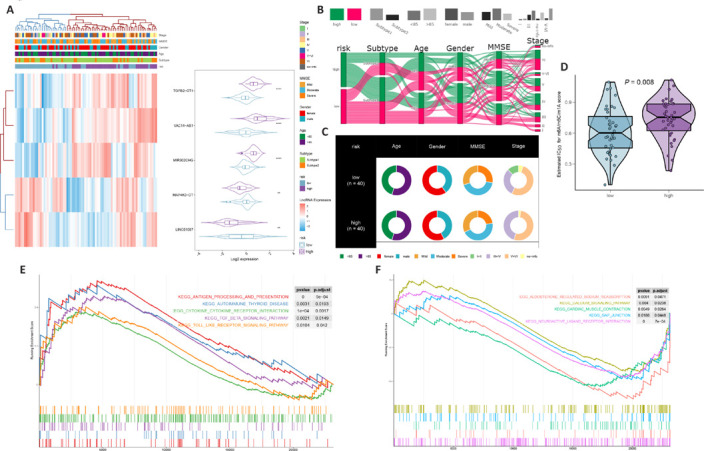
Construction of a risk model using the LASSO approach for predicting AD progression. (A) Heatmap (left) and violin plot (right) of the expression profile of the lncRNAs included in the risk model between low-risk and high-risk patients, annotated by age, sex, MMSE, Braak stage, and RNA methylation subtype. Horizontal lines indicate median and interquartile range. **P < 0.01, ****P < 0.0001, *vs.* low-risk group (Wilcoxon rank sum test). (B) Sankey diagram showing the relationships among risk score, subtype, age, sex, MMSE, and Braak stage. (C) Pie charts showing the differences in clinical features between low-risk and high-risk patients with AD. (D) Comparison of RNA methylation scores between low-risk and high-risk patients with AD. Horizontal lines indicate median and interquartile range. (E, F) GSEA of upregulated (E) and downregulated (F) key pathways in high-risk patients. AD: Alzheimer’s disease; GSEA: Gene set enrichment analysis; lncRNAs: Long non-coding RNAs; MMSE: Mini-Mental State Examination.

To thoroughly determine the correlation between risk score and AD, a risk evaluation model was developed, dividing 80 AD samples into low-risk and high-risk categories. The MALRs TGFB2-OT1, VAC14-AS1, and MIR302CHG were upregulated in the high-risk cohort, whereas MAP4K3-DT and LINC01007 exhibited significantly higher expression in the low-risk group (**Figure 11A**). Sankey diagram and pie chart analyses indicated that most patients in subtype 1 were classified within the high-risk category and typically presented with a more advanced stage of AD compared with their low-risk counterparts. No significant differences were noted in sex, age, or MMSE scores between the two risk groups (**Figure 11B** and **C**). Furthermore, a higher RNA methylation score (*P* = 0.008, *Cohen’s* *d* = 0.805) distinguished the high-risk group from the low-risk group. GSEA demonstrated that the high-risk group predominantly exhibited increased methylation of genes in immune and cytokine-mediated pathways, including antigen processing, autoimmune responses, cytokine-receptor interactions, transforming growth factor-β, and Toll-like receptor signaling (**Figure 11D**). The highly methylated regulatory pathways in the low-risk group included sodium reabsorption, calcium signaling, cardiac muscle contraction, gap junction functionality, and ligand–receptor interactions (**Figure 11E** and **F**), confirming the distinctions in methylation patterns and molecular pathway involvement identified in our prior analyses.

### Differences in the immune microenvironment and candidate drugs between the low- and high-risk groups

**Figure 12:**
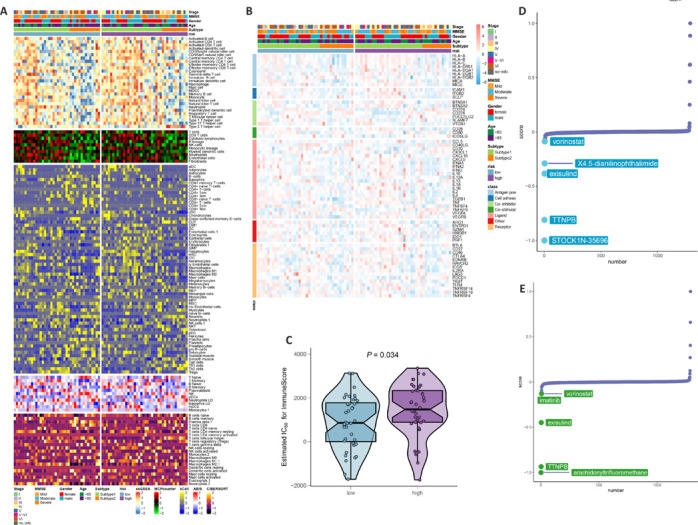
Immune characteristics of and predicted effective drugs in the risk model subgroups. (A) Heatmap of the expression profiles of infiltrated immune cells based on the ssGSEA, MCPcounter, xCell, ABIS, and ESTIMATE algorithms between low-risk and high-risk patients with AD. (B) Heatmap showing the expression profile of immunoregulatory subgroup genes between low-risk and high-risk patients with AD, annotated by age, sex, MMSE, and Braak stage. (C) Comparison of immune score between low-risk and high-risk patients with AD. Horizontal lines represent median and interquartile range (Wilcoxon rank sum test). (D, E) CMap analysis exhibiting the top five potential therapeutic drugs for low-risk (D) and high-risk (E) patients. ABIS: Amputee Body Image Scale; AD: Alzheimer’s disease; CMap: Connectivity map; MCPcounter: Microenvironment Cell Populations counter; ssGSEA: single sample gene set enrichment analysis; xCell: gene signature deconvolution method.

Changes in the immune microenvironment are crucial factors in AD development and progression. Therefore, we aimed to evaluate the immune microenvironment profiles of the high-risk and low-risk groups. We observed notably lower infiltration levels of various immune cell subsets in the low-risk group as opposed to the high-risk group (**Figure 12A**). To determine the disparity in immune regulation capability between the two risk groups, the expression levels of immunomodulatory genes were analyzed in both cohorts. Most of the upregulated immune regulatory genes were found in the high-risk group and were accompanied by a higher immune score (*P* = 0.034, *Cohen’s d* = 0.381; **Figure 12B** and **C**). These results suggest a stronger immune response in the high-risk group, which may be beneficial for immunotherapy.

Finally, using CMap analysis, potential therapeutic drugs for the low-risk and high-risk groups were investigated. As the CMap score decreased, the ability of the drug to treat AD improved. The five drugs exhibiting the lowest CMap scores in the low-risk group included vorinostat, X4.5-dianilinophthalimide, exisulind, tetrahydro-tetramethyl-naphthalenyl-propenyl benzoic acid (TTNPB), and STOCK1N-35696 (**Figure 12D**). By contrast, the most efficient drugs for the high-risk group were vorinostat, imatinib, exisulind, TTNPB, and arachidonyltrifluoromethane. Notably, STOCK1N-35696 and arachidonyltrifluoromethane demonstrated the lowest CMap scores in the low-risk and high-risk groups, respectively (**Figure 12E**). This finding implies their potential therapeutic efficacy for patients with varying levels of AD risk.

## Discussion

Owing to the heterogeneity of AD, patients exhibit varying degrees of treatment response and diverse clinical outcomes, resulting in limited progress in the individualization of AD treatment. Investigating the diversity of AD facilitates a more comprehensive understanding of the ailment and aids in the formulation of more efficacious therapeutic approaches. In this study, we meticulously assessed RNA methylation modification levels in patients with AD using advanced transcriptomic and scRNA-seq analyses. The expression levels of lncRNA methylation regulators were confirmed by qRT-PCR. Distinct heterogeneous subtypes were proposed, and their consistency was validated using an independent external dataset. Clinical attributes, biological functions, pathways, and immune microenvironment differences between the subtypes were characterized. In addition, three machine learning techniques (LASSO, RF, and XGBoost) were employed to identify signature MALRs. A prognostic model based on these signature MALRs was then developed, followed by assessment of molecular features, the immune microenvironment, and potential therapeutic drugs, offering targeted treatment strategies for patients with AD with varying risk profiles. Collectively, our findings shed light on the molecular diversity of AD through the innovative lens of RNA methylation, paving the way for personalized therapeutic approaches.

Our transcriptomic analysis highlighted significant alterations in RNA methylation regulators within AD brain tissue. These changes point to potential reprogramming of RNA methylation that could be a fundamental mechanism in AD pathogenesis. The identified regulators, such as IGF2BP1 and METTL3, have been previously implicated in neuronal function and disease processes, validating our findings within the broader context of AD research (Guo et al., 2022; Tang et al., 2023; Yin et al., 2023). Experimental validation of the identified regulators and therapeutic candidates in preclinical models is essential to confirm their biological relevance and potential as therapeutic targets.

Our results demonstrate a coordinated shift in the expression of these regulators, suggesting system-level dysregulation of RNA methylation. Consistent with the transcriptomics results, notably enhanced RNA methylation was detected in AD cells compared with those in the control group. Recent studies have indicated a significant correlation between RNA methylation and immune cell differentiation and activation (Chao et al., 2021; Grenov et al., 2021; Song et al., 2021). The landscape of RNA methylation in AD-infiltrating immune cells remains elusive. The single-cell analysis performed in this study deepened our understanding of RNA methylation by identifying specific immune cell subsets, particularly T cells, B cells, and NK cells, as exhibiting increased RNA methylation in AD. This cellular specificity implies that RNA methylation could be crucial to immune cell dysregulation, a phenomenon that is gaining recognition in the context of neurodegenerative diseases (McMillan et al., 2023; Tassinari et al., 2023). This study underscores the essential function of the immune system in AD progression and points to the promise of therapies that focus on immune-related mechanisms. Heterogeneity in clinical outcomes is usually determined by distinct molecular features. Using WGCNA, we identified 15 MALRs that were utilized to classify patients with AD into two distinct RNA methylation subtypes, according to a consensus clustering algorithm. Functional enrichment analysis demonstrated that subtype 1 was intensively linked to immune-related functions and pathways, whereas subtype 2 was mainly driven by metabolism, suggesting that the distinct molecular features between the two subtypes may lead to differences in prognosis. Therefore, to further explore individualized treatment for AD patients, we assessed the immune profile of each patient, including immune cell infiltrations, immune score, and expression of immune checkpoint inhibitors. Activated CD8^+^ T cells are the principal agents and direct effectors that cause neuronal dysfunction in AD brains (Stojić-Vukanić et al., 2020; Unger et al., 2020). AD symptoms can be exacerbated or alleviated based on the subsets of infiltrating CD4^+^ T cells. Pro-inflammatory subsets such as Th1 and Th17 T cells are the primary sources of pro-inflammatory cytokines that can weaken endothelial integrity and trigger astrocyte accumulation, thus leading to the production of beta-amyloid. Anti-inflammatory subsets such as Th2 and Treg T cells inhibit inflammation and modulate Th1 and Th17 T cell function (Dai et al., 2021). Furthermore, certain traditional immune checkpoint inhibitors, including programmed death ligand 1 (also known as CD274) and cytotoxic T lymphocyte antigen-4, serve as critical targets for immunotherapeutic approaches. Elevated levels of these molecules indicate an increase in the potential of treatment efficacy (Gou et al., 2020; Hosseini et al., 2020). In this study, subtype 1 was considered an immune subtype because it was characterized by a larger number of infiltrating immune cells, a higher immune score, and abundant enrichment of immunomodulatory genes, revealing that better immune responses and efficacy can be achieved in subtype 1. The prognostic significance of these lncRNAs is supported by their association with immune cell infiltration and immune regulatory gene expression, suggesting a direct link between RNA methylation and immune response modulation in AD.

Early identification and accurate diagnosis of AD play a crucial role in detecting high-risk populations and developing effective preventive measures. In this study, five characteristic MALRs—LINC01007, MAP4K3-DT, MIR302CHG, VAC14-AS1, and TGFB2-OT1—were determined on the basis of machine learning algorithms. Bioinformatics analysis revealed that LINC01007 participates in AD-related competing endogenous RNA networks and can serve as a biomarker for AD (Cao et al., 2019). MAP4K3-DT, VAC14-AS1, and MIR302CHG are newly discovered lncRNAs with unknown roles in disease progression. TGFB2-OT1, an emerging lncRNA originating from the 3’ untranslated region of TGFB2, can worsen atherosclerosis-related injury by modulating autophagy within vascular endothelial cells (Huang et al., 2015; Ma et al., 2016). Nevertheless, the association between these five lncRNAs and AD development and progression remains unclear. Our ROC curve analysis, nomogram, calibration curve, DCA, and validation in external datasets showed that these five MALRs can predict AD. It is anticipated that our findings will help establish a new theoretical foundation for understanding the involvement of these five MALRs in AD onset and progression.

Risk scores were calculated, and a risk model was constructed utilizing these five MALRs. Significantly, most patients in the high-risk group were classified as subtype 1. Extensive examination of the molecular pathways and the immune microenvironment differences between the low-risk and high-risk groups revealed considerable elevation in the expression levels of specific immune cells and immune checkpoint inhibitors in the high-risk group compared with the low-risk group, aligning with the previously observed subtype classification outcomes. This finding underscores the importance of personalized treatment strategies and the need for further investigation into the mechanisms by which these drugs modulate RNA methylation and immune responses in AD.

Another consideration is that, because of the heterogeneity of AD, some patients exhibit high sensitivity to particular drugs, while others may suffer side effects from the same drugs. Therefore, we attempted to predict effective drugs for patients with Alzheimer’s disease with varying risk profiles utilizing our differential gene expression data and the CMap database. The results demonstrated that the low-risk and high-risk groups shared common drugs, including vorinostat, exisulind, and TTNPB. Among these candidate drugs, vorinostat has been shown to induce vasodilation of the rat aorta through nitric oxide-induced activation of hemoglobin-containing guanylate cyclase, thus exerting anti-cancer abilities (Kenny et al., 2020). Exisulind represents a new category of medication that exhibits suppresses growth and enhances programmed cell death of cancer cells, thus impacting tumorigenesis (Goluboff, 2001; Whitehead et al., 2003). TTNPB, a retinoic acid analog, promotes neuronal differentiation by boosting the functions of retinoic acid receptor α and retinoic acid receptor γ (Das and Pethe, 2021). We determined that STOCK1N-35696 and arachidonyltrifluoromethane are the most effective drugs for the low-risk and high-risk groups, respectively. A previous study showed that arachidonyltrifluoromethane, a trifluoromethyl analogue of celecoxib, can suppress the release of pro-inflammatory cytokines and reduce neuronal axonal damage (Di Penta et al., 2013), suggesting that reduced inflammation mitigates neurodegeneration and cognitive decline. In addition, arachidonic acid derivatives, including arachidonyltrifluoromethane, are lipophilic molecules (Tsuji, 2005), suggesting that arachidonyltrifluoromethane may be able to cross the blood–brain barrier, although this requires further experimental validation. Unfortunately, whether arachidonyltrifluoromethane can indirectly affect RNA methylation by altering the expression of enzymes involved in RNA methylation or affecting the stability of RNA methylation regulators, thereby indirectly affecting RNA methylation, is largely unknown. Detailed reports regarding STOCK1N-35696 are currently lacking but, based on its identification through CMap, we speculate that it may target pathways involving cellular stress responses and inflammation. Future studies should utilize preclinical models to investigate how these compounds affect AD pathology, including their ability to cross the blood–brain barrier and their interactions with RNA methylation and immune pathways.

The present study has unique aspects that set it apart from similar research in the field. First, integrating machine learning with RNA sequencing and single-cell analysis provides a novel perspective on RNA methylation in AD, a dimension that is often overlooked in conventional studies. Second, the development of an RNA methylation score using diverse computational methods offers a more detailed understanding of the immune microenvironment in AD. Third, identifying MALRs using sophisticated machine learning algorithms represents a pioneering step towards precision medicine for AD. Fourth, constructing a risk model and nomogram, along with exploring the immune microenvironment and therapeutic predictions, provides actionable insights for clinical decision-making.

This study has several limitations. The retrospective nature of the analysis and the reliance on existing public datasets may have introduced selection bias and limited the generalizability of the findings, especially given the small cohort size and potential inconsistencies in data collection. The use of machine learning models, while insightful, carries the risk of overfitting, highlighting the need for further validation in larger, diverse populations. Additionally, while *in vivo* experiments were conducted, more functional studies are required to clarify the mechanistic links between RNA methylation and immune regulation in AD. Future research should focus on conducting prospective, multi-center studies, integrating multi-omics approaches, and validating therapeutic targets like lncRNAs in clinical trials to better understand and treat AD.

In conclusion, in this study we described the landscape of RNA methylation in patients with AD at the transcriptomic and single-cell sequencing levels. The altered expression of RNA methylation regulators in AD-treated rat neurons was validated by qRT-PCR analysis. Furthermore, on the basis of lncRNAs associated with RNA methylation, RNA methylation-related molecular subtypes and a risk model were proposed to accurately predict AD progression and immunological characteristics. These findings provide novel insight into AD heterogeneity based on RNA methylation and enable customization of personalized treatment methods for patients with AD.

## Additional files:

***Additional Figure 1:*** *Heatmap depicting the age-related changes in RNA methylation regulators between control and Alzheimer’s disease samples.*

Additional Figure 1 Heatmap depicting the age-related changes in RNA methylation regulators between control and Alzheimer’s disease samples.

***Additional Figure 2:*** *Heatmap (A) and violin plots (B) of immune cell infiltration between control and Alzheimer’s disease subjects.*

Additional Figure 2 Heatmap (A) and violin plots (B) of immune cell infiltration between control and Alzheimer’s disease subjects.
Horizontal lines represent median and interquartile range. ^*^P < 0.05, ^**^P < 0.01, ^***^P < 0.001, ^****^P < 0.0001 (Wilcoxon rank sum test). ns: Not significant.

***Additional Figure 3:*** *Single-cell level identification of RNA methylation scores across different cell subtypes within brain tissue.*

Additional Figure 3 Single-cell level identification of RNA methylation scores across different cell subtypes within brain tissue.
(A, B) UMAP plots depicting multiple cell cluster (A) and cell subtype (B) color-coded for clarity. (C) Heatmap showing the relative expression of specific marker genes within eight distinct cell subtypes. Genes with high or low expression are displayed in red or blue, respectively. (D) UMAP plots depicting the distribution of RNA methylation score across eight distinct cell subtypes in brain tissues. (E) Violin plots showing the differences in RNA methylation score across eight distinct cell subtypes based on the ssgsea algorithm. Horizontal lines represent median and interquartile range. UMAP: Uniform manifold approximation and projection.

***Additional Figure 4:*** *Identification of RNA methylation subtypes of Alzheimer’s disease patients using consensus clustering approach in GSE48350.*

Additional Figure 4 Identification of RNA methylation subtypes of Alzheimer’s disease patients using consensus clustering approach in GSE48350.
(A-E) Consensus clustering matrix when k equals 2-6. (F) Consensus cumulative distribution function curves when k equals 2-6. (G) Relative variations in cumulative distribution function delta area curves when k equals 2-6. (H) Consensus score of each RNA methylation subtypes when k equals 2-6. Different k values are represented by the numbers on the x-axis.

***Additional Figure 5:*** *Validation of RNA methylation subtypes of Alzheimer’s disease patients in GSE5281.*

Additional Figure 5 Validation of RNA methylation subtypes of Alzheimer’s disease patients in GSE5281.
(A-E) Consensus clustering matrix when k equals 2-6. (F) Consensus cumulative distribution function curves when k equals 2-6. (G) Relative variations in cumulative distribution function delta area curves when k equals 2-6. (H) t-SNE diagram classified subtype1 and sybtype2 patients. (I) Consensus score of each RNA methylation subtypes when k equals 2-6. Different k values are represented by the numbers on the x-axis.

***Additional Figure 6:*** *Differentially expressed gene screening between RNA methylation subtypes 1 and 2.*

Additional Figure 6 Differentially expressed gene screening between RNA methylation subtypes 1 and 2.
(A, B) Heatmap (A) and volcano plot (B) showing up- and down-regulation of differentially expressed gene expression levels.

***Additional Figure 7:*** *The differences in immune infiltration between RNA methylation subtypes.*

Additional Figure 7 The differences in immune infiltration between RNA methylation subtypes.
(A-E) Violin plots quantification of the expression profiles of infiltrated immune cells based on the ssGSEA (A), MCPcounter (B), xCell (C), ABIS (D), and CIBERSORT (E) algorithms between subtypes 1 and 2 patients. Horizontal lines represent median and interquartile range. ^*^P < 0.05, ^**^P < 0.01, ^***^P < 0.001, ^****^P < 0.0001, vs. subtype 1 group (Wilcoxon rank sum test). ns: Not significant.

***Additional Figure 8:*** *Characterization of the differences in metabolic (A) and immune (B) phenotypes across various brain cell types.*

Additional Figure 8 Characterization of the differences in metabolic (A) and immune (B) phenotypes across various brain cell types.
Horizontal lines represent median and interquartile range.

***Additional Figure 9:*** *SHAP dependence plot exhibiting the top 10 characteristic methylation-associated long non-coding RNAs contributing to the XGBoost model.*

Additional Figure 9 SHAP dependence plot exhibiting the top 10 characteristic methylation-associated long non-coding RNAs contributing to the XGBoost model.
The SHAP dependency plot illustrates how individual features affect the model's output. A SHAP value above 0 for a feature indicates an increased risk of Alzheimer’s disease. SHAP: Shapley Additive exPlanation.

***Additional Table 1:*** *The detailed information for gene datasets.*

***Additional Table 2:*** *Quantitative reverse transcription-polymerase chain reaction primers targeting the related genes.*

## Data Availability

*The datasets used and/or analyzed during the current study are available in the Gene Expression Omnibus (GEO, https://www.ncbi.nlm.nih.gov/geo/). All relevant data are within the paper and its Additional files.*
